# Positive Association between* ANKRD55* Polymorphism 7731626 and Dermatomyositis/Polymyositis with Interstitial Lung Disease in Chinese Han Population

**DOI:** 10.1155/2017/2905987

**Published:** 2017-04-02

**Authors:** Liubing Li, Si Chen, Xiaoting Wen, Qian Wang, Guanting Lv, Jing Li, Funing Yang, Fengchun Zhang, Yongzhe Li

**Affiliations:** ^1^Department of Rheumatology and Clinical Immunology, Peking Union Medical College Hospital, Chinese Academy of Medical Sciences & Peking Union Medical College, Key Laboratory of Rheumatology and Clinical Immunology, Ministry of Education, Beijing, China; ^2^Department of Clinical Laboratory, Beijing Anzhen Hospital, Capital Medical University, Beijing, China; ^3^Department of Blood Transfusion, Tangdu Hospital, The Fourth Military Medical University, Xi'an, China; ^4^Department of Medical Laboratory, The First Hospital of Jilin University, Changchun, China

## Abstract

Single nucleotide polymorphisms (SNPs) in* TNFSF4* and* ANKRD55 *genes have been shown to be associated with several autoimmune diseases, although whether these genes are susceptibility genes for dermatomyositis/polymyositis (DM/PM) has, to date, not been reported. This study aimed to investigate the potential associations of these SNPs with DM/PM in a Chinese Han population. Five SNPs in* TNFSF4 *(rs2205960, rs844644, and rs844648) and* ANKRD55 *(rs6859219, rs7731626) genes were genotyped using the SequenomMassArray system in 2297 Chinese individuals. In total, 1017 DM/PM patients and 1280 gender-matched healthy controls were genotyped. No significant associations were observed in DM/PM patients for the five SNPs analyzed. The association between SNPs and interstitial lung disease (ILD) was also investigated. Both DM-ILD (*P*_*c*_ = 0.030, OR = 0.65, 95% CI: 0.47–0.88) and DM/PM-ILD (*P*_*c*_ = 0.015, OR = 0.67, 95% CI: 0.51–0.87) exhibited a significant association with the rs7731626-A allele. Rs7731626-A was less frequently found in DM-ILD and DM/PM-ILD patients compared with healthy controls. This is the first study to demonstrate a positive association between* ANKRD55 *polymorphism and DM-ILD and DM/PM-ILD. A decreased frequency of rs7731626-A in DM-ILD and DM/PM-ILD patients suggests that the A variant may be protective against DM/PM-ILD.

## 1. Introduction

Dermatomyositis (DM) and polymyositis (PM) are autoimmune diseases that are most prevalent in women and the elderly [1], characterized by the shared features of skeletal muscle weakness, elevated serum creatine kinase levels, and inflammation in muscle biopsy [2, 3]. It has been reported that DM/PM prevalence ranges from 21.42 to 32.74/100,000 person-years (py) in the USA [1, 4, 5] and from 10 to 13/100,000 py in Japan with an increasing trend [6]. Though DM/PM most frequently affects the skin and muscles, it may also affect multiple organs, most notably, the lung. Interstitial lung disease (ILD) is the primary pulmonary manifestation, which is associated with a high morbidity and mortality in DM/PM patients [7–9]. It would, therefore, be advantageous to investigate the biomarkers associated with the development of ILD in patients with DM/PM. To date, several genetic [10–12] and environmental [13–16] factors have been proposed to contribute to the development of DM/PM. However, these identified predisposing factors are unable to completely account for the pathogenesis of DM/PM.

Studies have reported that autoimmune diseases share a number of human leukocyte antigen (HLA) and non-HLA genes [12, 17–19]. A genome-wide association study (GWAS) revealed DM shares genetic susceptibility factors with rheumatoid arthritis (RA), multiple sclerosis (MS), systemic lupus erythematosus (SLE), and other autoimmune diseases [12]. Recent studies identified that single nucleotide polymorphisms (SNPs) of* TNFSF4* (rs2205960, rs844644, and rs844648) and* ANKRD55* (rs6859219, rs7731626) also shared susceptibility loci for RA [20–22], MS [17], and SLE [23, 24]. The association of the five SNPs with the diseases discussed is summarized in Table  S1 (see Table S1 in the Supplementary Material available online at https://doi.org/10.1155/2017/2905987).* TNFSF4* encodes the ligand for* TNFRSF4*, and this receptor-ligand pair can activate CD4+ T cells [25]. The* TNFSF4-TNFRSF4* interaction has been shown to contribute to the induction of antitumor immunity [26] and the inhibition of allergic responses [27, 28].* ANKRD55* encodes ankyrin repeat domain-containing protein 55, which mediates protein-protein interactions. A recent report has revealed that* ANKRD55 *can be detected in resting CD4+ T cells and monocytes and may have possible relevance to autoimmune diseases (http://www.amazonia.transcriptome.eu) [29]. However, the exact function of* ANKRD55 *remains unknown. In order to elucidate whether the five genetic polymorphisms of* TNFSF4* and* ANKRD55 *are associated with DM/PM in a Chinese Han population, we performed the current study including 1017 DM/PM patients and 1280 matched healthy controls.

## 2. Materials and Methods

### 2.1. Subjects

The current study was a multicenter case-control study, approved by the Ethics Committee of the Peking Union Medical College Hospital, China. Patients with DM/PM fulfilled the Bohan and Peter criteria [30] and were recruited between February 2013 and June 2015. A total of 1017 DM/PM patients including 569 patients from the Peking Union Medical College Hospital, along with 448 patients from another 21 centers in China, were enrolled. The sera from 659 recruited patients were analyzed for anti-Jo-1 antibody using QUANTA Lite Jo-1-ELISA assay (Inova Diagnostics, San Diego, CA, USA). Patients with ILD were identified with high-resolution chest computed tomography (HRCT). In addition, 1280 gender-matched healthy individuals from the Peking Union Medical College Hospital were recruited in this study. 287 DM/PM patients from Peking Union Medical College Hospital were followed up for 3 yrs. All participants were from the Chinese Han population and provided signed informed consent.

### 2.2. Genotyping

We collected 2 ml peripheral blood from each participant in an ethylenediaminetetraacetic acid- (EDTA-) coated tube. DNA was extracted from peripheral white blood cell using genomic DNA kits (Tiangen, Beijing, China) and stored at −80°C until use. The genotyping of the five SNPs from* TNFSF4* and* ANKRD55* genes was performed using SequenomMassArray system (San Diego, CA, USA) according to the manufacturer's protocol. Primers for polymerase chain reaction (PCR) and locus-specific single-base extension were designed by MassArray Assay Design 4.0 (Sequenom). Briefly, all DNA samples were transferred to a 384-well plate. After multiplex PCR amplications, the products were used for locus-specific single-base extension reaction. Afterwards, the products were desalted and transferred onto a 384-element SpectroCHIParray (Sequenom). Allele detection was conducted by matrix-assisted laser desorption/ionization-time-of-flight mass spectrometry (MALDI-TOF MS). Finally, MassArrayTyper 4.0 software was used to analyze the resultant mass spectrograms and genotype data.

### 2.3. Statistical Analysis

The Chi-square (*χ*^2^) test was used to assess whether each SNP in the control populations conformed to Hardy-Weinberg equilibrium (HWE). Any SNPs that deviated from the HWE (*P* < 0.05 in the controls) would be excluded in the analysis. The differences in allelic and genotypic frequencies between patients and controls were calculated by *χ*^2^ test. The risk allele frequency for each SNP between DM/PM patients with ILD and controls was calculated. The odds ratio (OR) and 95% confidence interval (95% CI) of the association were calculated; *P* values < 0.05 (adjusted by Bonferroni correction) were considered to be statistically significant. The logistic regression models (additive, dominant, and recessive models) were used to further analyze the genotype frequencies. For the association analysis between the five SNPs and the three subsets (DM, PM, or overall DM/PM versus controls), statistical analysis was performed using PLINK v1.07 (Shaun Purcell, Boston, MA, USA) [31]. The association study for the five SNPs and the presence of ILD were performed according to the results of the following comparisons: patients with ILD versus patients without ILD, patients with ILD versus controls, and patients without ILD versus controls.

## 3. Results

### 3.1. Clinical Characteristics of Participants

The demographics and clinical features of enrolled DM/PM patients and healthy controls were displayed in Table 1. A total of 1017 DM/PM patients (25.7% male, 74.3% female; mean age 46.1 ± 15.2) were recruited, including 654 DM patients and 363 PM patients. Among these patients, 390 of 654 patients had DM and 195 of 363 patients had PM complicated by ILD. In total, 585 of 1017 patients had DM/PM complicated by ILD, while 432 did not. Of 659 DM/PM patients who were assessed for anti-Jo-1 antibody, 115 patients (61 DM patients and 54 PM patients) were positive for anti-Jo-1 antibody. During the 3-year follow-up, 14 DM/PM patients developed new malignant tumors, including six patients with lung cancer, three with breast cancer, two with ovarian cancer, one with liver cancer, one with nasopharynx cancer, and one with synovial sarcoma. The analysis of the five selected SNPs is shown in Table 2. All SNPs had call rates > 99% and followed HWE in the controls (*P*_*c*_ > 0.05, Table 2).

### 3.2. Association of the Five SNPs with DM, PM, or DM/PM in the Han Population

Five SNPs rs2205960, rs844644, rs844648, rs6859219, and rs7731626 were genotyped in 1017 DM/PM patients (DM, *n* = 654; PM, *n* = 363) and 1280 normal controls. The allelic and genotypic frequency distribution of the five SNPs are summarized in Table 3. No significant difference in allelic and genotypic frequencies was found between patients and controls (*P*_*c*_ > 0.05, Table 3). We further performed logistic regression analysis based on three genetic models (additive, dominant, and recessive models). None of the three genetic models showed any significant difference between patients and controls for the five SNPs (*P*_*c*_ > 0.05, Table 4).

### 3.3. Association between the Five SNPs and ILD Phenotype of DM/PM

To analyze the association between the five SNPs and ILD phenotype of DM/PM patients, the five SNPs of* TNFSF4* and* ANKRD55* were genotyped in 585 DM/PM patients with ILD (DM-ILD, *n* = 390; PM-ILD, *n* = 195). A significantly decreased frequency of SNP rs7731626-A was observed in DM-ILD (6.54% in DM-ILD, 9.77% in the controls, *P*_*c*_ = 0.030, OR = 0.65, 95% CI: 0.47–0.88, Table 5) and DM/PM-ILD patients (6.75% versus 9.77%, *P*_*c*_ = 0.015, OR = 0.67, 95% CI: 0.51–0.87, Table 5) as compared to healthy controls. No significant association was observed between SNP rs7731626-A and PM patients with ILD. The clinical significance of SNP rs7731626-A for DM-ILD and DM/PM-ILD still needs further study. In addition, no significant association was detected between the other four SNPs and patients with ILD or without ILD when comparing patients with ILD versus patients without ILD, patients with ILD versus controls, or patients without ILD versus controls (*P*_*c*_ > 0.05, Table 5).

## 4. Discussion

In this multiple-center, large-sample case-control study, we investigated the associations of* TNFSF4* (rs2205960, rs844644, and rs844648) and* ANKRD55* (rs6859219, rs7731626) polymorphisms with the susceptibility to DM/PM in a Chinese Han population. Our results demonstrated that* ANKRD55 *polymorphism (rs7731626) was significantly associated with DM-ILD as well as DM/PM-ILD. A significant decrease in the frequency of rs7731626-A allele in DM-ILD and DM/PM-ILD patients compared to controls may suggest that this allele may play a protective role against the development of ILD. Notably, this was the first study to demonstrate that the* ANKRD55* polymorphism was associated with Chinese DM/PM patients with ILD.

ILD is a common complication of DM/PM and progression of ILD is a leading course of mortality [7–9]. Several gene polymorphisms have been revealed to be associated with DM/PM patients with ILD, including* STAT4* rs7574865 polymorphism [32] and* TNFAIP3* rs2230926 and rs5029939 polymorphisms [33]. However, these identified susceptibility genes do not fully account for the genetic pathogenesis of DM/PM-ILD.* ANKRD55 *has been shown to be inducible following inflammatory stimuli, and its expression may also increase susceptibility to inflammation in patients [29]. An increase in the protein expression of* ANKRD55 *in autoimmune encephalomyelitis mice suggests that this molecule may act as a disease biomarker. Although the precise function of* ANKRD55* is still unknown, a link between genetic variants of* ANKRD55* and autoimmune diseases may exist. At loci* ANKRD55*, rs7731626 is located in immune cell enhancer regions [34]. To date, few studies have examined the association of SNP rs7731626 of* ANKRD55* with different diseases. Okada et al. reported that rs7731626-G was a risk factor for the development of RA by comparing 12,841 RA patients with 33,416 healthy controls in a European population [20]. In our presented study, our data demonstrated rs7731626 was negatively associated with DM-ILD and DM/PM-ILD in Chinese Han patients, whereby the protective A allele was the minor allele in these patients. Our results indicated that DM/PM-ILD may share a common genetic locus with RA. However, no association was found between DM/PM and SNP rs6859219 of* ANKRD55*, which has been shown to be related to MS [17, 35] and RA [21, 22] (Table  S1).

*TNFSF4* with its receptor* TNFRSF4 *promotes CD4+ T cells activation as a potent costimulatory signal [36]. Recent studies have demonstrated that polymorphisms of* TNFSF4* (rs2205960, rs844644, and rs844648) are associated with SLE [23, 24, 37, 38] and systemic sclerosis (SSc) [39] (Table  S1). To explore whether SNPs rs2205960, rs844644, and rs844648 are also associated with DM/PM, we conducted the present study. Our data showed no statistically significant association existed between* TNFSF4 *polymorphisms and DM/PM, suggesting the pathogenesis of DM/PM may differ from SLE and SSc. Additional studies in different ethnic groups should be performed in the future to further investigate the association of rs2205960, rs844644, and rs844648 with DM/PM.

There are, however, some limitations to our study. The current case-control study revealed that the minor allele (A) of rs7731626 was less frequent in DM-ILD and DM/PM-ILD than in controls (Table 5). We hypothesize that the A variant may be protective for DM/PM-ILD. However, in order to decide if this result is robust enough to also be clinically significant, multiple studies with different sample sizes are needed in the future study. Whether this variant affects the properties of ANKRD55 protein in a way which is compatible with this hypothesis requires further confirmation. In addition, we only investigated the association of rs7731626 with DM-ILD or DM/PM-ILD in the Han Chinese population. Due to ethnic genetic differences, further investigations are required to confirm the association of* ANKRD55* genetic variants with DM-ILD or DM/PM-ILD in Caucasian and African patients.

## 5. Conclusions

This study was the first study to elucidate that a rare variant within the* ANKRD55* gene (rs7731626) is protective in DM-ILD and DM/PM-ILD in a Chinese Han population. Further studies are needed to investigate the association of* ANKRD55 *genetic variants with DM-ILD or DM/PM-ILD in different ethnic groups from variable geographical regions.

## Supplementary Material

The associations of the five SNPs with different diseases.

## Figures and Tables

**Table 1:** Clinical features of DM/PM patients and healthy controls.

	Cases		Controls	
	Number/total	%	Number/total	%
Mean age	46.1 ± 15.2		41.8 ± 12.7	
Male	261/1017	25.7	165/1280	12.9
Female	756/1017	74.3	1115/1280	87.1
DM	654/1017	64.3	—	—
PM	363/1017	35.7	—	—
ILD	585/1017	57.5	—	—
DM with ILD	390/1017	38.3	—	—
PM with ILD	195/1017	19.2	—	—
Anti-Jo-1 antibody	115/659	17.5	—	—
DM with anti-Jo-1 antibody	61/438	13.9	—	—
PM with anti-Jo-1 antibody	54/221	24.4	—	—
Malignancy	14/287	4.9	—	—
Lung cancer	6/14	42.9	—	—
Breast cancer	3/14	21.4	—	—
Ovarian cancer	2/14	14.3	—	—
Liver cancer	1/14	7.1	—	—
Nasopharynx cancer	1/14	7.1	—	—
Synovial sarcoma	1/14	7.1	—	—

DM = dermatomyositis; PM = polymyositis; ILD = interstitial lung disease.

**Table 2:** The information of five selected SNPs.

Gene	SNP	Chromosome	Position	Function class	Allele	MAF in CHB	MAF	*P*_*c*_ for HWE	Call rate
						(dbSNP)			(%)
*TNFSF4*	rs2205960	1	173222336	NA	G > T	0.198	0.265	0.655	100
*TNFSF4*	rs844644	1	173240356	Intron	A > C	0.384	0.445	NS	99.96
*TNFSF4*	rs844648	1	173254724	Intron	G > A	0.384	0.453	NS	100
*ANKRD55*	rs6859219	5	56142753	Intron	C > A	0.012	0.005	NS	100
*ANKRD55*	rs7731626	5	56148856	Intron	G > A	NA	0.089	NS	100

The information was gathered from dbSNP database in NCBI. SNPs = single nucleotide polymorphisms; MAF = minor allele frequency; CHB = Han Chinese in Beijing, China; *P*_*c*_ = *P* value corrected by Bonferroni method; HWE = Hardy-Weinberg equilibrium; NA = not available; NS = not significant.

**Table 3:** Allele and genotype frequencies of the five SNPs in DM/PM patients and healthy controls.

Gene	SNP	Groups	Allele (%)		OR (95% CI)	*P*_*c*_	Genotype (%)			*χ* ^2^	*P*_*c*_
*TNFSF4*	rs2205960		T	G			TT	TG	GG		
		DM	344 (26.30)	964 (73.70)	1.00 (0.86–1.16)	NS	43 (6.57)	258 (39.45)	353 (53.98)	0.30	NS
		PM	197 (27.13)	529 (72.87)	1.04 (0.87–1.26)	NS	21 (5.79)	155 (42.70)	187 (51.52)	0.59	NS
		DM/PM	541 (26.60)	1493 (73.40)	1.01 (0.89–1.16)	NS	64 (6.29)	413 (40.61)	540 (53.10)	0.05	NS
		Controls	674 (26.33)	1886 (73.67)			78 (6.09)	518 (40.47)	684 (53.44)		

*TNFSF4*	rs844644		C	A			CC	CA	AA		
		DM	601 (45.95)	707 (54.05)	1.10 (0.96–1.26)	0.850	129 (19.72)	343 (52.45)	182 (27.83)	3.17	NS
		PM	325 (44.77)	401 (55.23)	1.05 (0.89–1.24)	NS	67 (18.46)	191 (52.62)	105 (28.93)	1.40	NS
		DM/PM	926 (45.53)	1108 (54.47)	1.08 (0.96–1.21)	0.995	196 (19.27)	534 (52.51)	287 (28.22)	3.53	0.855
		Controls	1116 (43.63)	1442 (56.37)			243 (19.00)	630 (49.26)	406 (31.74)		

*TNFSF4*	rs844648		A	G			AA	AG	GG		
		DM	607 (46.41)	701 (53.59)	1.08 (0.94–1.23)	NS	129 (19.72)	349 (53.36)	176 (26.91)	5.53	0.315
		PM	332 (45.73)	394 (54.27)	1.05 (0.89–1.24)	NS	68 (18.73)	196 (53.99)	99 (27.27)	4.07	0.655
		DM/PM	939 (46.17)	1095 (53.83)	1.07 (0.95–1.20)	NS	197 (19.37)	545 (53.59)	275 (27.04)	7.50	0.115
		Controls	1141 (44.57)	1419 (55.43)			263 (20.55)	615 (48.05)	402 (31.41)		

*ANKRD55*	rs6859219		A	C			AA	AC	CC		
		DM	5 (0.38)	1303 (99.62)	0.75 (0.27–2.11)	NS	0 (0.00)	5 (0.76)	649 (99.24)	NA	NA
		PM	5 (0.69)	721 (99.31)	1.36 (0.48–3.82)	NS	0 (0.00)	5 (1.38)	358 (98.62)	NA	NA
		DM/PM	10 (0.49)	2024 (99.51)	0.97 (0.42–2.21)	NS	0 (0.00)	10 (0.98)	1007 (99.02)	NA	NA
		Controls	13 (0.51)	2547 (99.49)			0 (0.00)	13 (1.02)	1267 (98.98)		

*ANKRD55*	rs7731626		A	G			AA	AG	GG		
		DM	99 (7.57)	1209 (92.43)	0.76 (0.59–0.96)	0.120	5 (0.76)	89 (13.61)	560 (85.63)	4.93	0.425
		PM	58 (7.99)	668 (92.01)	0.80 (0.60–1.08)	0.735	1 (0.28)	56 (15.43)	306 (84.30)	NA	NA
		DM/PM	157 (7.72)	1877 (92.28)	0.77 (0.63–0.95)	0.075	6 (0.59)	145 (14.26)	866 (85.15)	6.14	0.23
		Controls	250 (9.77)	2310 (90.23)			16 (1.25)	218 (17.03)	1046 (81.72)		

SNPs = single nucleotide polymorphisms; DM = dermatomyositis; PM = polymyositis; OR = odds ratio; CI = confidence interval; *P*_*c*_ = *P* value corrected by Bonferroni method; *χ*^2^ = Chi-square test; NS = not significant; NA = not available.

**Table 4:** Analysis of the five SNPs based on additive, dominant, and recessive model.

Gene	SNP	Allele	Group	Additive model		Dominant model		Recessive model	
				*P*_*c*_	OR (95% CI)	*P*_*c*_	OR (95% CI)	*P*_*c*_	OR (95% CI)
*TNFSF4*	rs2205960	T	DM	NS	1.00 (0.86–1.17)	NS	0.98 (0.81–1.18)	NS	1.09 (0.74–1.59)
			PM	NS	1.04 (0.86–1.26)	NS	1.08 (0.86–1.36)	NS	1.09 (0.74–1.59)
			DM/PM	NS	1.02 (0.89–1.16)	NS	1.01 (0.86–1.20)	NS	1.04 (0.74–1.46)

*TNFSF4*	rs844644	C	DM	0.830	1.10 (0.96–1.26)	0.385	1.21 (0.98–1.48)	NS	1.05 (0.83–1.33)
			PM	NS	1.05 (0.89–1.24)	NS	1.14 (0.89–1.48)	NS	1.05 (0.83–1.33)
			DM/PM	0.965	1.08 (0.96–1.22)	0.340	1.18 (0.99–1.42)	NS	1.02 (0.83–1.26)

*TNFSF4*	rs844648	A	DM	NS	1.08 (0.94–1.23)	0.205	1.24 (1.01–1.53)	NS	0.95 (0.75–1.20)
			PM	NS	1.05 (0.89–1.24)	0.655	1.22 (0.94–1.58)	NS	0.95 (0.75–1.20)
			DM/PM	NS	1.07 (0.95–1.20)	0.115	1.24 (1.03–1.48)	NS	0.93 (0.76–1.14)

*ANKRD55*	rs6859219	A	DM	NS	0.75 (0.27–2.12)	NS	0.75 (0.27–2.12)	NA	NA
			PM	NS	1.36 (0.48–3.84)	NS	1.36 (0.48–3.84)	NA	NA
			DM/PM	NS	0.97 (0.42–2.22)	NS	0.97 (0.42–2.22)	NA	NA

*ANKRD55*	rs7731626	A	DM	0.135	0.76 (0.60–0.97)	0.155	0.75 (0.58–0.97)	NS	0.61 (0.22–1.67)
			PM	0.760	0.81 (0.60–1.08)	NS	0.83 (0.61–1.14)	0.700	0.61 (0.22–1.67)
			DM/PM	0.085	0.78 (0.63–0.96)	0.145	0.78 (0.62–0.97)	0.575	0.47 (0.18–1.20)

SNPs = single nucleotide polymorphisms; *P*_*c*_ = *P* value corrected by Bonferroni method; OR = odds ratio; CI = confidence interval; DM = dermatomyositis; PM = polymyositis; NS = not significant; NA = not available.

**Table 5:** Association of the five SNPs and DM/PM patients with ILD.

Disease	Group	rs2205960 *(TNFSF4)*			rs844644 *(TNFSF4)*			rs844648 *(TNFSF4)*			rs6859219 *(ANKRD55)*			rs7731626 *(ANKRD55)*		
		Risk allele T (%)	*P*_*c*_	OR (95% CI)	Risk allele C (%)	*P*_*c*_	OR (95% CI)	Risk allele A (%)	*P*_*c*_	OR (95% CI)	Risk allele A (%)	*P*_*c*_	OR (95% CI)	Risk allele A (%)	*P*_*c*_	OR (95% CI)
DM	P versus N	24.49 versus 28.98	0.350	0.79 (0.62–1.02)	45.38 versus 46.78	NS	0.95 (0.76–1.18)	46.41 versus 46.40	NS	1.00 (0.80–1.25)	0.51 versus 0.19	NS	2.72 (0.30–24.37)	6.54 versus 9.09	0.435	0.70 (0.46–1.06)
	P versus C	24.49 versus 26.33	NS	0.91 (0.75–1.09)	45.38 versus 43.63	NS	1.07 (0.91–1.26)	46.41 versus 44.57	NS	1.08 (0.92–1.27)	0.51 versus 0.51	NS	1.01 (0.33–3.11)	6.54 versus 9.77	**0.030**	0.65 (0.47–0.88)
	N versus C	28.98 versus 26.33	NS	1.14 (0.93–1.41)	46.78 versus 43.63	0.920	1.14 (0.94–1.37)	46.40 versus 44.57	NS	1.08 (0.89–1.30)	0.19 versus 0.51	NS	0.37 (0.05–2.85)	9.09 versus 9.77	NS	0.92 (0.67–1.28)

PM	P versus N	27.18 versus 27.08	NS	1.01 (0.72–1.40)	45.64 versus 43.75	NS	1.08 (0.80–1.45)	46.92 versus 44.35	NS	1.11 (0.83–1.49)	0.51 versus 0.89	NS	0.57 (0.10–3.45)	7.18 versus 8.93	NS	0.79 (0.46–1.35)
	P versus C	27.18 versus 26.33	NS	1.04 (0.82–1.33)	45.64 versus 43.63	NS	1.09 (0.88–1.34)	46.92 versus 44.57	NS	1.10 (0.89–1.36)	0.51 versus 0.51	NS	1.01 (0.23–4.49)	7.18 versus 9.77	0.515	0.71 (0.48–1.07)
	N versus C	27.08 versus 26.33	NS	1.04 (0.80–1.34)	43.75 versus 43.63	NS	1.01 (0.80–1.26)	44.35 versus 44.57	NS	0.99 (0.79–1.25)	0.89 versus 0.51	NS	1.77 (0.50–6.23)	8.93 versus 9.77	NS	0.91 (0.61–1.35)

DM/PM	P versus N	25.38 versus 28.24	0.750	0.86 (0.71–1.05)	45.47 versus 45.60	NS	0.99 (0.83–1.19)	46.58 versus 45.60	NS	1.04 (0.87–1.24)	0.51 versus 0.46	NS	1.11 (0.31–3.94)	6.75 versus 9.03	0.285	0.73 (0.53–1.01)
	P versus C	25.38 versus 26.33	NS	0.95 (0.81–1.12)	45.47 versus 43.63	NS	1.08 (0.94–1.24)	46.58 versus 44.57	NS	1.08 (0.94–1.25)	0.51 versus 0.51	NS	1.01 (0.38–2.66)	6.75 versus 9.77	**0.015**	0.67 (0.51–0.87)
	N versus C	28.24 versus 26.33	NS	1.10 (0.93–1.31)	45.60 versus 43.63	NS	1.08 (0.93–1.27)	45.60 versus 44.57	NS	1.04 (0.89–1.22)	0.46 versus 0.51	NS	0.91 (0.30–2.80)	9.03 versus 9.77	NS	0.92 (0.70–1.20)

Bold values indicate statistical significance (*P*_*c*_ < 0.05); SNPs = single nucleotide polymorphisms; DM = dermatomyositis; PM = polymyositis; ILD = interstitial lung disease; *P*_*c*_ = *P* value corrected by Bonferroni method; OR = odds ratio; CI = confidence interval; Group P: patients with ILD; Group N: patients without ILD; Group C: healthy controls; Group P (DM: *n* = 390; PM: *n* = 195; DM/PM: *n* = 585); Group N (DM: *n* = 264; PM: *n* = 168; DM/PM: *n* = 432); Group C (*n* = 1280).
