# Supplementary material for: Positive Association between ANKRD55 Polymorphism 7731626 and Dermatomyositis/Polymyositis with Interstitial Lung Disease in Chinese Han Population
Source: Biomed Res Int. 2017 Apr 2;2017:2905987. doi: 10.1155/2017/2905987 (PMC5392395; doi:10.1155/2017/2905987)
Supplement: Supplementary file 1 — The associations of the five SNPs with different diseases. [file 2905987.f1.docx]

Table S1. The association of the five SNPs with different diseases.

| Author and year | Disease | Ethnicity | Cases | Healthy controls | Risk allele | *P* | OR (95% CI) | *Pc* | Article type | PMID |
| --- | --- | --- | --- | --- | --- | --- | --- | --- | --- | --- |
| Okada 2014 | RA | European | 12841 | 33416 | G | 8.2×10^(-23) | 1.21 (1.17-1.26) | — | GWAS meta-analysis | 24390342 |
| Lill 2013 | MS | Germany and France | 6974 | 6875 | C | 1.0×10^(-5) | 1.15 (1.08-1.23) | — | article | 23315543 |
| Alloza 2012 | MS | Spainish | 2895 | 2942 | C | 2.3×10^(-9) | 1.35 (1.22-1.49) | — | article | 22130326 |
| Zhernakova 2011 | RA | European | 8384 | 22175 | C | 4.31×10^(-9) | 0.78 | — | GWAS meta-analysis and replication | 21383967 |
| Stahl 2010 | RA | European | 12307 | 28975 | C | 9.6×10^(−12) | — | — | GWAS meta-analysis and replication | 20453842 |
| Lu 2016 | BD | Han Chinese | 394 | 606 | T | 0.637 | 1.050 (0.856-1.288) | NS | article | 27872495 |
| Lu 2016 | Vogt–Koyanagi–Harada | Han Chinese | 395 | 606 | T | 0.054 | 1.217 (0.996-1.487) | NS | article | 27872495 |
| Chua 2016 | SLE | Chinese | 336 | 359 | T | 0.05 | 1.27 (1.00-1.61) | 0.2 | article | 27519474 |
| Chua 2016 | SLE | Malay | 109 | 114 | T | 0.91 | 1.03 (0.64-1.56) | NA | article | 27519474 |
| Chua 2016 | SLE | Indian | 31 | 36 | T | 4×10^(-3) | 3.16 (1.41-7.05) | 0.02 | article | 27519474 |
| Chua 2016 | SLE | Malaysian | 476 | 509 | T | — | 1.42 (0.90-2.23) | 0.132 | article | 27519474 |
| Zhou 2013 | SLE | Chinese | 814 | 722 | T | 4.2×10^(-5) | — | — | article | 23936824 |
| Manku 2013 | SLE | African-American Gullah | 1680 | 2170 | T | 7.20×10^(-5) | 1.48 (1.2-1.67) | — | article | 23874208 |
| Manku 2013 | SLE | East Asian | 1500 | 1396 | T | 1.18×10^(-10) | 1.43 (1.32-1.54) | — | article | 23874208 |
| Manku 2013 | SLE | European | 3432 | 3640 | T | 5.61×10^(-15) | 1.33 (1.26-1.40) | — | article | 23874208 |
| Manku 2013 | SLE | Hispanic | 1348 | 717 | T | 1.70×10^(-10) | 1.56 (1.42-1.69) | — | article | 23874208 |
| Sun 2013 | pSS | Han Chinese | 555 | 597 | T | 0.25 | 1.11 （0.93-1.34） | NS | article | 23635951 |
| Kong 2013 | pSS | Han Chinese | 250 | 393 | T | 0.031 | 1.309 (1.025-1.673) | NS | article | 23622253 |
| Kong 2013 | PBC | Han Chinese | 221 | 393 | T | 0.044 | 1.299 (1.007-1.675) | NS | article | 23622253 |
| Coustet 2012 | SSc | French white | 1011 | 1011 | T | 0.06 | 1.15 (0.99-1.33) | 0.3 | article | 22422496 |
| Coustet 2012 | anticentromere antibodies positive-SSc | French white | 368 | 1011 | T | 0.0015 | 1.37 (1.12-1.66) | 0.015 | article | 22422496 |
| Zhou 2012 | SLE | Chinese | 804 | 722 | T | 2.82×10^(-5) | 1.39 (1.19-1.62) | 1.19×10^(-4) | article | 21905002 |
| Gourh 2010 | SSc | Caucasian | 698 | 1059 | T | 0.01 | 1.24 (1.1-1.5) | 0.019 | article | 19778912 |
| Chang 2009 | SLE | Hong Kong Chinese | 949 | 1042 | T | 2.41×10^(-4) | 1.296 (1.13-1.49) | — | article | 19357697 |
| Han 2009 | SLE | Han Chinese | 4199 | 8255 | A | 2.53×10^(-32) | 1.46 (1.37-1.56) | — | article | 19838193 |
| Graham 2008 | SLE | UK | 424 | 642 | A | 0.03 | — | — | article | 18059267 |
| Lu 2016 | BD | Han Chinese | 394 | 606 | A | 0.698 | 0.965 (0.807-1.155) | NS | article | 27872495 |
| Lu 2016 | Vogt–Koyanagi–Harada | Han Chinese | 395 | 606 | A | 0.184 | 1.130 (0.944-1.135) | NS | article | 27872495 |
| Manku 2013 | SLE | African-American Gullah | 1680 | 2170 | A | 0.91 | 1.00 (0.88-1.13) | — | article | 23874208 |
| Manku 2013 | SLE | East Asian | 1500 | 1396 | A | 2.56×10^(-11) | 0.71 (0.67-0.77) | — | article | 23874208 |
| Manku 2013 | SLE | European | 3432 | 3640 | A | 1.04×10^(-7) | 0.84 (0.78-0.91) | — | article | 23874208 |
| Manku 2013 | SLE | Hispanic | 1348 | 717 | A | 3.03×10^(-5) | 0.75 (0.62-0.89) | — | article | 23874208 |
| Castillo 2011 | SSc | European | 2912 | 2946 | A | — | 0.93 (0.86-1.00) | 0.07 | article | 21187296 |
| Castillo 2011 | limited cutaneous SSc | European | 1653 | 2946 | A | — | 0.91 (0.83-0.99) | 0.04 | article | 21187296 |
| Castillo 2011 | anticentromere antibodies positive-SSc | European | 856 | 2946 | A | — | 0.90 (0.80-1.00) | 0.049 | article | 21187296 |
| Gourh 2010 | SSc | Caucasian | 698 | 1059 | A | 0.01 | 0.84(0.7-0.97) | 0.019 | article | 19778912 |
| Delgado-Vega 2009 | SLE | Argentina | 278 | 355 | A | 1.59×10^(-3) | 0.69 (0.55-0.87) | 1.99×10^(-3) | article | 19092840 |
| Delgado-Vega 2009 | SLE | Germany | 259 | 322 | A | 0.7691 | 0.97 (0.76-1.22) | 0.7691 | article | 19092840 |
| Delgado-Vega 2009 | SLE | Italy | 258 | 223 | A | 5.68×10^(-4) | 0.64 (0.49-0.82) | 2.84×10^(-3) | article | 19092840 |
| Delgado-Vega 2009 | SLE | Spain | 517 | 901 | A | 0.6064 | 1.04 (0.89-1.21) | 0.6064 | article | 19092840 |
| Graham 2008 | SLE | UK | 424 | 642 | A | 6.8×10^(-5) | — | — | article | 18059267 |
| Chua 2016 | SLE | Chinese | 336 | 359 | A | — | 1.20 (0.97-1.48) | NS | article | 27519474 |
| Chua 2016 | SLE | Malay | 109 | 114 | A | — | 0.94 (0.65-1.37) | NS | article | 27519474 |
| Chua 2016 | SLE | Indian | 31 | 36 | A | — | 1.51 (0.76-2.98) | NS | article | 27519474 |
| Chua 2016 | SLE | Malaysian | 476 | 509 | A | — | 1.15 (0.97-1.38) | 0.114 | article | 27519474 |
| Manku 2013 | SLE | African-American Gullah | 1680 | 2170 | — | 0.34 | 1.05 (0.95-1.15) | — | article | 23874208 |
| Manku 2013 | SLE | East Asian | 1500 | 1396 | — | 5.17×10^(-8) | 1.33 (1.23-1.43) | — | article | 23874208 |
| Manku 2013 | SLE | European | 3432 | 3640 | — | 2.59×10^(-13) | 1.28 (1.22-1.35) | — | article | 23874208 |
| Manku 2013 | SLE | Hispanic | 1348 | 717 | — | 3.07×10^(-8) | 1.44 (1.31-1.57) | — | article | 23874208 |
| Sun 2013 | pSS | Han Chinese | 555 | 597 | A | 0.068 | 1.17 （0.99-1.38） | 0.613 | article | 23635951 |
| Castillo 2011 | SSc | European | 2940 | 2977 | A | — | 1.07 (1.00-1.15) | 0.07 | article | 21187296 |
| Castillo 2011 | limited cutaneous SSc | European | 1673 | 2977 | A | — | 1.1 (1.01-1.20) | 0.04 | article | 21187296 |
| Castillo 2011 | anticentromere antibodies positive-SSc | European | 860 | 2977 | A | — | 1.12 (1.01-1.25) | 0.049 | article | 21187296 |
| Coustet 2012 | SSc | French white | 1009 | 1004 | A | 0.086 | 1.12 (0.99-1.3) | 0.43 | article | 22422496 |
| Coustet 2012 | anticentromere antibodies positive-SSc | French white | 367 | 1004 | A | 0.0054 | 1.27 (1.07-1.5) | 0.054 | article | 22422496 |
| Gourh 2010 | SSc | Caucasian | 698 | 1059 | A | 0.03 | 116 (1.01-1.3) | 0.038 | article | 19778912 |
| Chang 2009 | SLE | Hong Kong Chinese | 949 | 1042 | A | 2.47×10^(-3) | 1.215 (1.07-1.38) | — | article | 19357697 |
| Delgado-Vega 2009 | SLE | Argentina | 278 | 355 | A | 3.87×10^(-3) | 0.72 (0.58-0.90) | 3.87×10^(-3) | article | 19092840 |
| Delgado-Vega 2009 | SLE | Germany | 259 | 322 | A | 3.26×10^(-2) | 1.29 (1.02-1.63) | 0.0544 | article | 19092840 |
| Delgado-Vega 2009 | SLE | Italy | 258 | 223 | A | 1.14×10^(-2) | 1.40 (1.08-1.82) | 1.14×10^(-2) | article | 19092840 |
| Delgado-Vega 2009 | SLE | Spain | 517 | 901 | A | 0.4074 | 0.94 (0.80-1.10) | 0.5093 | article | 19092840 |
| Graham 2008 | SLE | UK | 424 | 642 | T | 7.0×10^(-3) | — | — | article | 18059267 |

SNPs = single nucleotide polymorphisms; OR = odds ratio; CI = confidence interval; *Pc* = *P* value corrected by Bonferroni method; RA = rheumatoid arthritis; MS = multiple sclerosis; BD = Behcet's Disease; SLE = systemic lupus erythematosus; pSS = primary Sjogren’s syndrome; PBC = primary biliary cirrhosis; SSc = systemic sclerosis; GWAS = Genome-wide association study; NS = not significant.
